# Identification of patients at risk for poor survival among those with normal skeletal muscle mass: a multicenter retrospective validation study in gastric cancer

**DOI:** 10.1007/s10120-026-01778-z

**Published:** 2026-07-06

**Authors:** Ryota Matsui, Kazuyoshi Yamamoto, Yoshiro Yukawa, Masayoshi Terayama, Yukinori Kurokawa, Daisuke Ichikawa, Yoshitomo Yanagimoto, Takashi Oshima, Souya Nunobe, Takashi Kamei, Hiroya Takeuchi, Yoshihiro Nabeya, Masaki Kaibori, Naoki Hiki, Hidetoshi Eguchi, Yuichiro Doki

**Affiliations:** 1 Department of Gastroenterological Surgery, The Cancer Institute Hospital of Japanese Foundation for Cancer Research, 3-8-31, Ariake, Koto-ku, Tokyo, 135-8550 Japan; 2 Department of Gastroenterological Surgery, Graduate School of Medicine, The University of Osaka, Suita, Japan; 3 Department of Gastroenterological Surgery, Osaka International Cancer Institute, Osaka, Japan; 4 First Department of Surgery, Faculty of Medicine, Graduate School of Medicine, University of Yamanashi, Kofu, Yamanashi Japan; 5 Department of Surgery, Toyonaka Municipal Hospital, Toyonaka, Japan; 6 Department of Gastrointestinal Surgery, Kanagawa Cancer Center, Yokohama, Japan; 7 Department of Surgery, Tohoku University Graduate School of Medicine, Sendai, Japan; 8 Department of Surgery, Hamamatsu University School of Medicine, Hamamatsu, Japan; 9 Division of Esophago‑Gastrointestinal Surgery, Chiba Cancer Center, Chiba, Japan; 10 Department of Surgery, Kansai Medical University, Hirakata, Japan; 11 Department of Upper Gastrointestinal Surgery, School of Medicine, Kitasato University, Sagamihara, Japan

**Keywords:** Gastric cancer, Overall survival, Prognosis, Sarcopenia, Skeletal muscle mass

## Abstract

**Background:**

Although a low skeletal muscle mass index (SMI) leads to poor overall survival (OS) after gastrectomy in patients with gastric cancer, the cutoff value that predicts long-term survival in patients with normal SMI remains unclear. This study aimed to determine the cutoff SMI value for predicting OS in patients with normal SMI.

**Methods:**

This multicenter retrospective cohort study included patients with normal SMI who underwent radical gastrectomy for pStage I–III primary gastric cancer between January 2011 and December 2016. The patients were randomly divided at a 6:4 ratio into a training set, which examined the cutoff values for SMI, and a validation set, which validated the cutoff values. Patients were classified into high and low SMI groups according to the cutoff values. We compared OS between the high- and low-SMI groups using log-rank test and identified prognostic factors using Cox proportional hazards regression analysis.

**Results:**

Of the 2516 patients, 1389 (55.2%) and 1127 (44.8%) were classified into the training and validation sets. We examined the cutoff values of SMI, which were 44.3 cm^2^/m^2^ for men and 37.6 cm^2^/m^2^ for women. Both men and women in the low-SMI group had poorer OS than those in the high-SMI group. Multivariate analysis revealed that a low SMI was an independent adverse prognostic factor for OS (hazard ratio: 1.314, 95% confidence interval: 1.044–1.654, *P* = 0.020). These cutoff values were associated with lower cancer-specific survival.

**Conclusions:**

Among patients with normal SMI, those with a lower SMI had poorer long-term survival after radical gastrectomy.

## Introduction

Low skeletal muscle mass is a key factor associated with poor outcomes after gastrectomy in patients with gastric cancer. Systematic reviews have demonstrated that low skeletal muscle mass is not only a risk factor for postoperative complications but also a factor contributing to poor long-term survival [1–5]. As the patient population ages, the number of patients with low skeletal muscle mass is expected to increase. In recent years, low skeletal muscle mass has been shown to be associated with malnutrition [6], attracting greater attention as a target for nutritional interventions rather than just exercise. Consequently, the demand for preoperative evaluations using body composition analysis to identify intervention targets is expected to increase significantly.

However, the cutoff value predicting poor long-term survival in patients without low skeletal muscle mass remains unclear. Previous reports have shown that muscle mass decreases with weight loss in patients undergoing gastrectomy [7]. Furthermore, it is known that S-1, used for postoperative adjuvant chemotherapy, reduces muscle mass [8, 9]. Patients without preoperative sarcopenia who develop postoperative sarcopenia have poorer long-term survival [10]. However, the cutoff value associated with poor prognosis in patients with normal preoperative skeletal muscle mass remains unclear. Identifying this cutoff value would enable the identification of patients in the prefrailty or presarcopenia stages.

The objective of this study was to determine the cutoff value that predicts poor long-term survival among patients without low skeletal muscle mass. We previously reported the cutoff value for skeletal muscle mass that impairs overall survival (OS) in a Japanese multicenter retrospective cohort study and defined this as low skeletal muscle mass [11]. This study was a subgroup analysis focusing on patients without low skeletal muscle mass and examined the association between muscle mass and OS.

## Materials and methods

### Study design and population

This multicenter retrospective cohort study was conducted to evaluate the association between preoperative skeletal muscle mass index (SMI), assessed using computed tomography (CT), and clinical outcomes in patients with gastric cancer who underwent radical gastrectomy between January 2011 and December 2016. This study included 17 hospitals in Japan. Eligible patients were diagnosed with gastric or esophagogastric junction adenocarcinoma and agreed to participate in the study. Patients were excluded if they had received neoadjuvant chemotherapy or chemoradiotherapy, did not undergo preoperative CT examination, were diagnosed with a low SMI according to our report [11], or were considered unsuitable for enrollment at the discretion of the attending physician. Clinical data and prognostic outcomes were collected from each database. Tumor staging was determined based on the 15th edition of the Japanese Classification of Gastric Carcinoma [12].

All experimental protocols described in this study were approved by the institutional review board of each participating hospital prior to study initiation. This study adhered to the ethical guidelines of the Japan Ministry of Health, Labour and Welfare for Medical and Health Research Involving Human Subjects and conformed to the provisions of the Declaration of Helsinki. Information regarding the study was made public, and an opt-out recruitment method was applied to allow patients to decline participation. The study protocol was registered in the University Hospital Medical Information Network Clinical Trials Registry (UMIN000053115).

### Definition of SMI

Muscle mass was evaluated using preoperative SMI values. Skeletal muscle mass was measured on preoperative plain CT using a graphic analysis. SMI was measured as the cross-sectional area of all muscles at the level of the third lumbar vertebra (range, 29–150 Hounsfield units) and was calculated by dividing the area in one CT slice by the patient’s height (m^2^) [5, 13]. Patients diagnosed with a low SMI according to the cutoff value we calculated [11] were excluded from this study.

Patients without a low SMI were assigned to a training set to determine the cutoff values for preoperative SMI and a validation set to confirm the validity of the cutoff values. We determined the cutoff values according to sex for patients assigned to the training set. SMI values lower than the cutoff value were defined as relatively low SMI (r-low SMI) within the normal skeletal muscle mass range, whereas SMI values higher than the cutoff value were defined as a high SMI.

### Outcomes

The primary outcome was OS, defined as the time from surgery to death from any cause. The secondary outcomes included cancer-specific survival (CSS) and other-cause survival (OCS). CSS was defined as the time from surgery to death due to gastric cancer. OCS was defined as the time from surgery to death from causes other than gastric cancer, including other cancers.

Postoperative complications were defined as complications of grade ≥ 2 according to the Clavien–Dindo classification (CD) occurring within 30 days after surgery, total postoperative complications, and severe complications of CD grade ≥ 3 [14].

### Statistical analysis

All statistical analyses were performed using the EZR software (Saitama Medical Center, Jichi Medical University, Saitama, Japan). Patients were retrospectively registered at multiple institutions and were divided into a training set and an independent validation set at the facility level. To construct these sets, institutions were randomly allocated so that approximately 40% of the total sample size was assigned to the validation set and the remaining 60% to the training set, while ensuring a sufficient number of men and women patients in each set. In the training set, sex-specific cutoff values for SMI were determined using restricted cubic spline modeling within a Cox proportional hazards framework. For men and women separately, the association between SMI and OS was modeled using Cox regression with SMI entered as a restricted cubic spline with four knots. Because extremely low SMI values (< 20th percentile) were sparse and yielded unstable estimates, and the hazard ratio curve tended to flatten at higher SMI values, the search for the optimal cutoff was restricted a priori to the range between the 20th and 50th percentiles of the SMI distribution. The restricted cubic spline curves were visually inspected to confirm that the point at which the clinical risk began to increase most clearly was contained within this pre-specified range. Within this interval, the SMI value corresponding to the maximum estimated hazard ratio was selected as the optimal sex-specific cutoff. Patients were divided into high- and r-low SMI groups based on the cutoff values, and the long-term survival in the validation set was compared between the two groups. We used the log-rank test in the Kaplan–Meier survival analysis for OS and Cox proportional hazards regression for univariate and multivariate analyses to identify prognostic factors and calculate hazards ratios (HRs). The factors included in the multivariate analysis were selected based on those previously reported to be associated with OS, and were included regardless of the results of the univariate analysis. The concordance index (C-index) was calculated to evaluate the discriminative ability of the predictive model using the concordance function in the survival package in R. A higher C-index value indicates greater predictive accuracy. Comparisons between the two groups were conducted using the Mann–Whitney U test for continuous variables and the chi-square test or Fisher's exact test for categorical variables. Statistical significance was set at *p* < 0.05.

## Results

### Study flowchart

**Fig.1:**
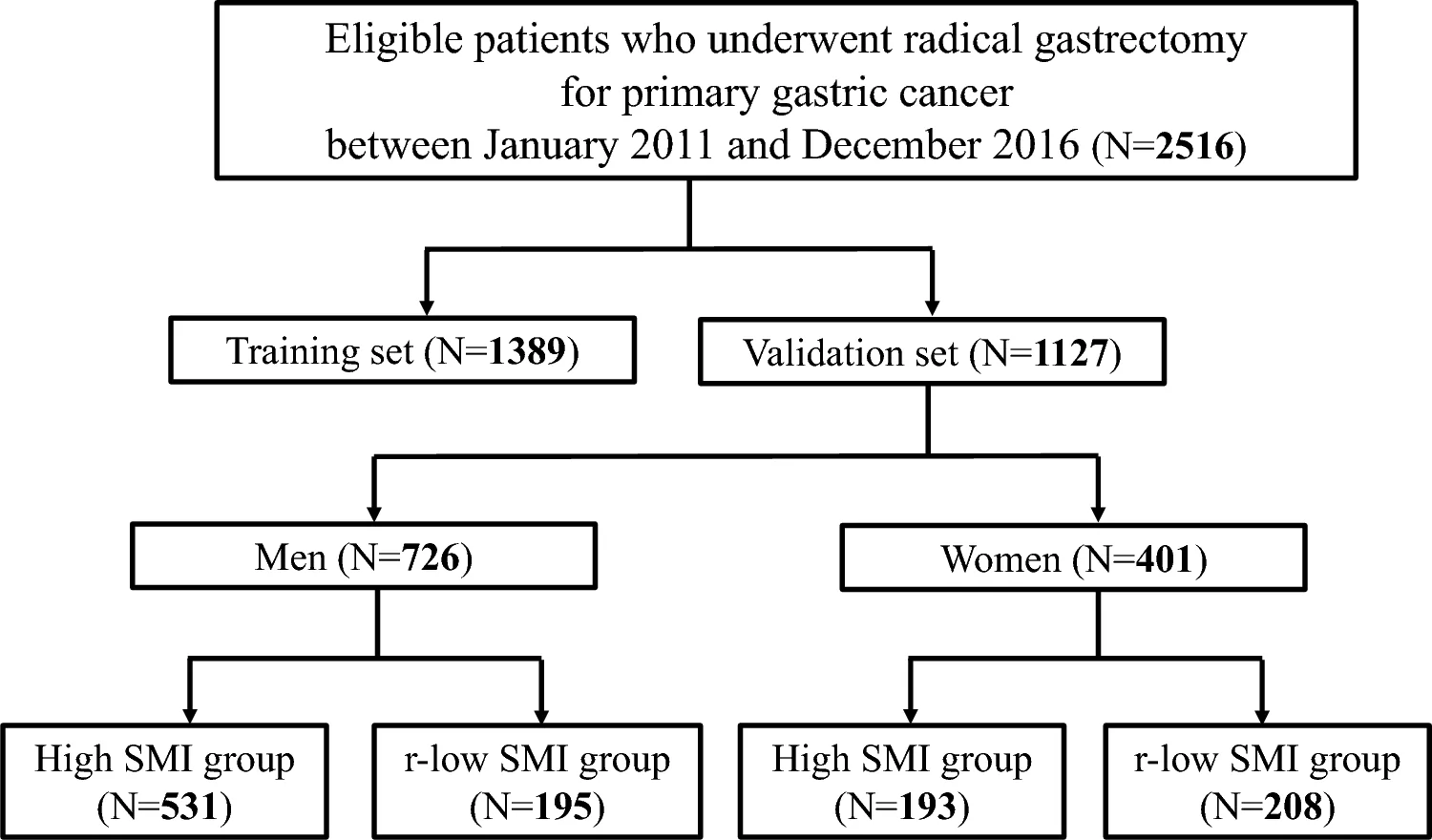
Flowchart of this study

**Fig.2:**
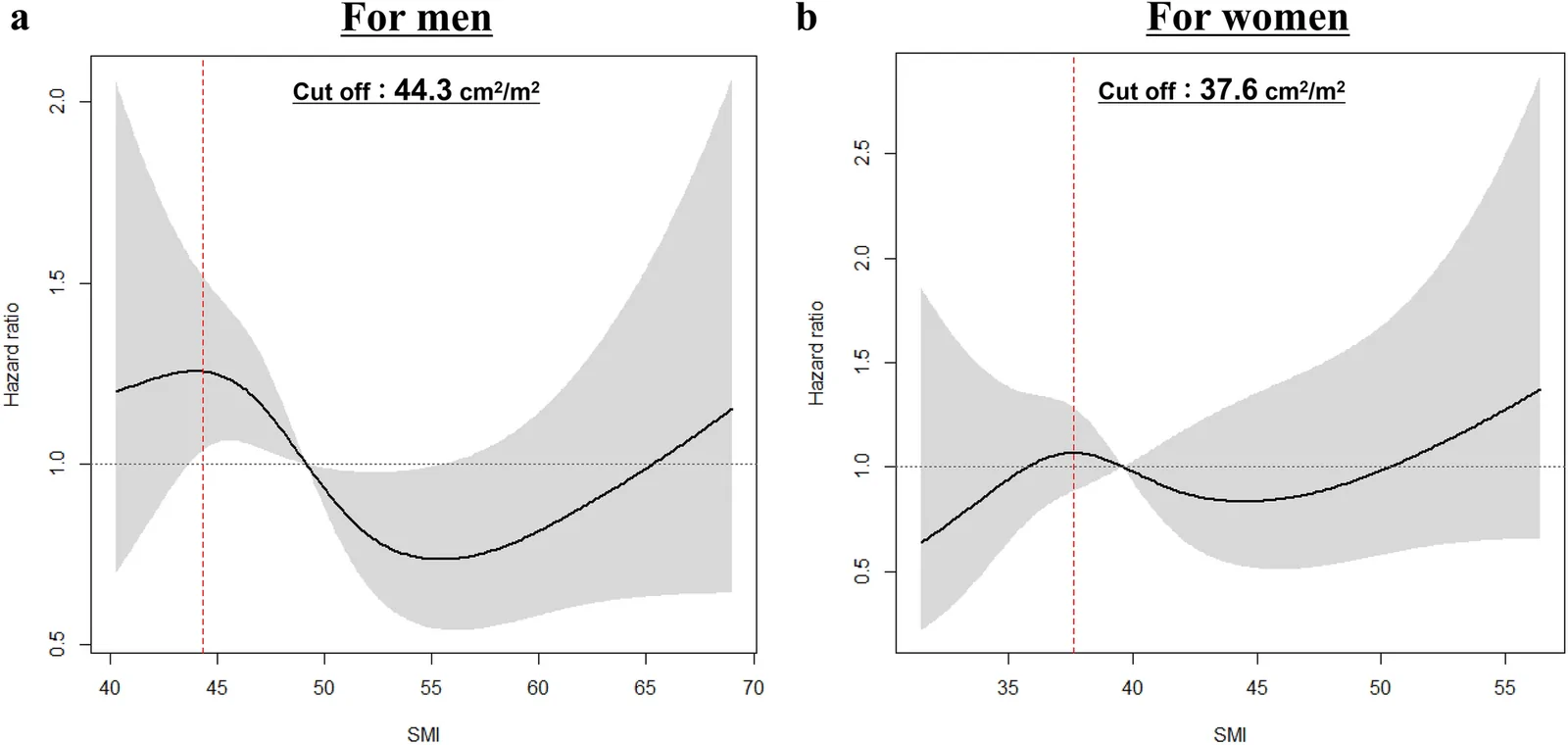
Restricted cubic spline curves for the association between SMI and overall survival in the training set, with derived sex-specific cutoff values: **a** for men, **b** for women

Of the 2516 patients, 1389 (55.2%) and 1127 (44.8%) were classified into the training and validation sets, respectively (Fig. 1). We used the training set to determine the cutoff values of preoperative SMI, which were 44.3 cm^2^/m^2^ for men (Fig. 2a) and 37.6 cm^2^/m^2^ for women (Fig. 2b). Therefore, we defined high SMI as ≥ 44.3 cm^2^/m^2^ in men and ≥ 37.6 cm^2^/m^2^ in women, and used the validation set to classify patients into high SMI and r-low SMI groups for men and women, respectively (Fig. 1).

### Patient characteristics

Table 1 shows the characteristics of all patients and those in the validation set by sex according to preoperative SMI values. In men, the r-low SMI group was older (*P* < 0.001), and had a lower BMI (*P* < 0.001). Among women, the r-low SMI group had a lower BMI (*P* < 0.001), and a lower incidence of postoperative complications (*P* = 0.029).

**Table 1:** Patient characteristics

	All patients	Validation set					
	N = 2516	Men (N = 726)			Women (N = 401)		
		High SMI group (N = 531)	r-low SMI group (N = 195)	*P* value	High SMI group (N = 193)	r-low SMI group (N = 208)	*P* value
Sex Men	1657 (65.9%)						
Women	859 (34.1%)						
Age (years), Median (IQR)	67.0 [60.0, 75.0]	66.0 [59.0, 73.5]	70.0 [63.0, 77.0]	< 0.001	66.0 [54.0, 76.0]	66.0 [58.0, 74.0]	0.864
BMI (kg/m^2^), Median (IQR)	23.0 [21.0, 24.9]	24.3 [22.9, 26.0]	22.3 [21.1, 24.0]	< 0.001	23.1 [21.3, 25.1]	21.1 [19.7, 22.6]	< 0.001
ASA-PS 1	784 (31.2%)	235 (44.3%)	81 (41.5%)	0.567	106 (54.9%)	123 (59.1%)	0.489
2	1512 (60.1%)	272 (51.2%)	102 (52.3%)		81 (42.0%)	77 (37.0%)	
3	164 (6.5%)	24 (4.5%)	12 (6.2%)		5 (2.6%)	8 (3.8%)	
4	2 (0.1%)	0 (0%)	0 (0%)		1 (0.5%)	0 (0%)	
SMI (cm^2^/m^2^), Median (IQR)	45.7 [40.8, 51.1]	49.7 [46.8, 53.6]	42.5 [41.4, 43.4]	< 0.001	40.8 [39.4, 43.5]	34.4 [32.9, 35.8]	< 0.001
*Preoperative CRP (mg/dL)*							
Median (IQR)	0.09 [0.04, 0.17]	0.09 [0.04, 0.19]	0.08 [0.04, 0.22]	0.631	0.05 [0.04, 0.13]	0.04 [0.03, 0.10]	0.154
> 0.5 mg/dL	254 (10.1%)	43 (8.1%)	27 (13.8%)	0.025	21 (10.9%)	20 (9.6%)	0.527
*Preoperative albumin (g/dL)*							
Median (IQR)	4.10 [3.80, 4.40]	4.10 [3.90, 4.40]	4.10 [3.80, 4.30]	0.038	4.10 [3.80, 4.30]	4.00 [3.80, 4.30]	0.774
< 3.5 g/dL	210 (8.3%)	28 (5.3%)	18 (9.2%)	0.059	18 (9.3%)	16 (7.7%)	0.594
*Clinical stage*							
I	1761 (70.0%)	363 (68.4%)	136 (69.7%)	0.201	135 (69.9%)	139 (66.8%)	0.708
II	408 (16.2%)	86 (16.2%)	21 (10.8%)		24 (12.4%)	34 (16.3%)	
III	315 (12.5%)	76 (14.3%)	35 (17.9%)		29 (15.0%)	31 (14.9%)	
IVA	32 (1.3%)	6 (1.1%)	3 (1.5%)		5 (2.6%)	4 (1.9%)	
*Surgical approach*							
Laparoscopic surgery	1657 (65.9%)	330 (62.1%)	129 (66.2%)	0.340	129 (66.8%)	145(69.7%)	0.591
Open surgery	859 (34.1%)	201 (37.9%)	66 (33.8%)		64 (33.2%)	63 (30.3%)	
*Surgical procedure*							
DG	1626 (64.6%)	319 (60.1%)	139 (71.3%)	0.039	131 (67.9%)	138 (66.3%)	0.806
TG	626 (24.9%)	126 (23.7%)	31 (15.9%)		33 (17.1%)	35 (16.8%)	
PG	174 (6.9%)	55 (10.4%)	18 (9.2%)		13 (6.7%)	12 (5.8%)	
PPG	90 (3.6%)	31 (5.8%)	7 (3.6%)		16 (8.3%)	23 (11.1%)	
*Lymph node dissection*							
D1 +	1441 (57.3%)	338 (63.7%)	119 (61.0%)	0.544	121 (62.7%)	124 (59.6%)	0.540
D2	1075 (42.7%)	193 (36.3%)	76 (39.0%)		72 (37.3%)	84 (40.4%)	
Serosal invasion	320 (12.7%)	73 (13.7%)	28 (14.4%)	0.810	29 (15.0%)	28 (13.5%)	0.670
*Lymph node metastasis*							
N1	339 (13.5%)	72 (13.6%)	25 (12.8%)	0.652	27 (14.0%)	19 (9.1%)	0.593
N2	241 (9.6%)	47 (8.9%)	21 (10.8%)		14 (7.3%)	18 (8.7%)	
N3	207 (8.2%)	43 (8.1%)	17 (8.7%)		13 (6.7%)	22 (10.6%)	
*Pathological stage*							
I	1646 (65.4%)	346 (65.2%)	128 (65.6%)	0.606	131 (67.8%)	139 (66.8%)	0.877
II	443 (17.6%)	99 (18.6%)	30 (15.4%)		31 (16.1%)	33 (15.9%)	
III	427 (17.0%)	86 (16.2%)	37 (19.0%)		31 (16.1%)	36 (17.3%)	
*Histological type*							
Differentiated type	1318 (52.4%)	293 (55.2%)	122 (62.6%)	0.129	77 (39.9%)	77 (37.0%)	0.608
Undifferentiated type	1194 (47.5%)	238 (44.8%)	73 (37.4%)		116 (60.1%)	131 (63.0%)	
*Postoperative complications*							
Overall complications	544 (21.6%)	142 (26.7%)	48 (24.6%)	0.634	45 (23.3%)	30 (14.4%)	0.029
Severe complications	153 (6.1%)	45 (8.5%)	13 (6.7%)	0.537	9 (4.7%)	6 (2.9%)	0.433
Adjuvant chemotherapy	584 (23.3%)	125 (23.5%)	53 (27.2%)	0.312	44 (22.8%)	49 (23.6%)	0.906
Discontinuation	213 (8.5%)	37 (7.0%)	24 (12.3%)	0.042	8 (4.1%)	10 (4.8%)	0.342

*ASA-PS* American Society of Anesthesiologists-physical status, *BMI* body mass index, *CRP* C-reactive protein, *DG* distal gastrectomy, *IQR* interquartile range, *PG* proximal gastrectomy, *PPG* pylorus-preserving gastrectomy, *SMI* skeletal muscle mass index, *TG* total gastrectomy

### Long-term outcomes

**Fig.3:**
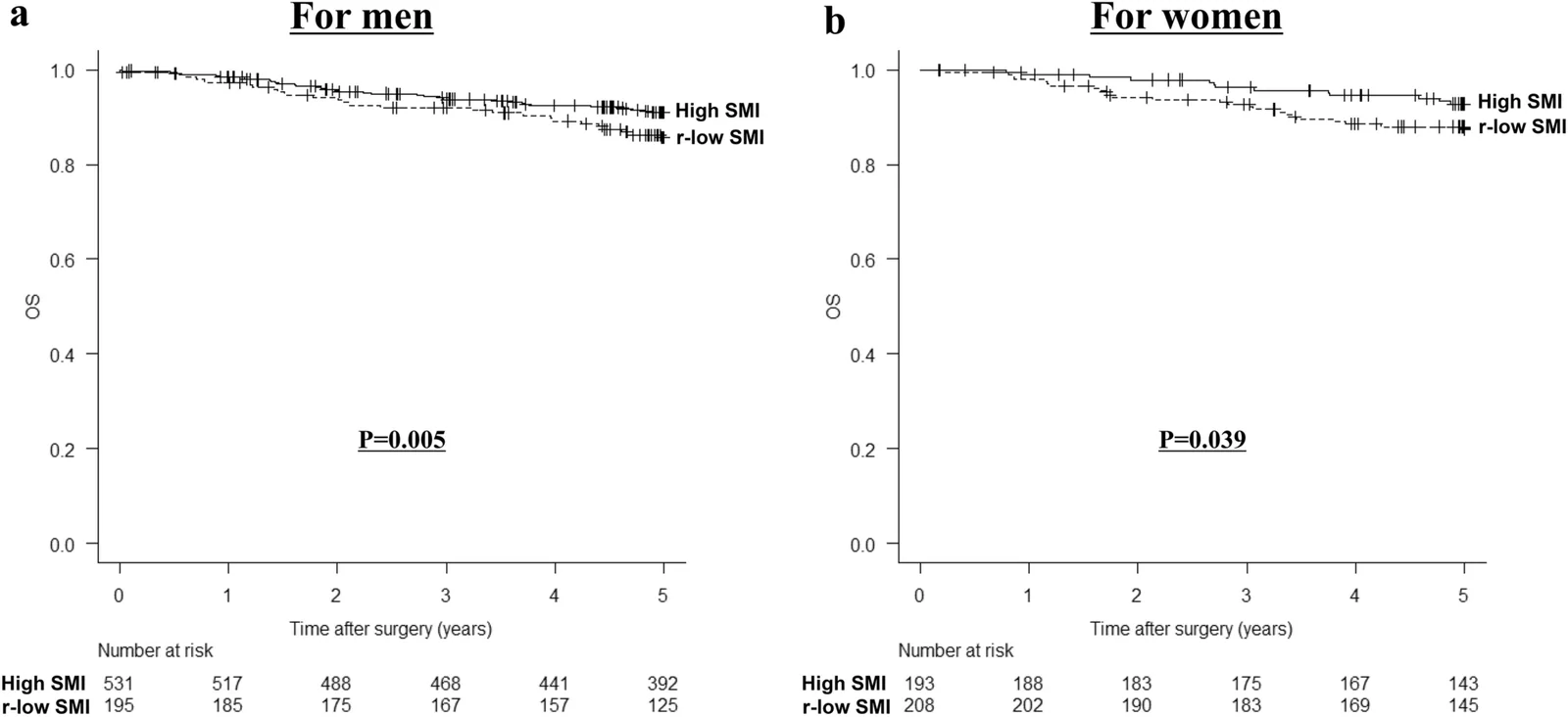
Overall survival by SMI in gastric cancer patients with normal muscle mass in the validation set: **a** for men, **b** for women

The median follow-up period was 62 months (interquartile range: 60–77 months). Figure 3 shows the OS according to the SMI in the validation set. In men, the OS of patients with r-low SMI was significantly worse than that of patients with a high SMI (*P* = 0.005; Fig. 3a). Similarly, in women, the OS of patients with r-low SMI was worse than that of patients with a high SMI (*P* = 0.039; Fig. 3b).

### Prognostic factors based on multivariate analysis

Table 2 shows the results of the multivariate analysis of OS in all patients. The r-low SMI was an independent poor prognostic factor (HR: 1.314, 95% confidence interval (CI): 1.044–1.654, *P* = 0.020).

**Table 2:** Results of analysis of prognostic factors for overall survival in all patients

Variables	Univariate analysis			Multivariate analysis		
	HR	95%CI	*P* value	HR	95%CI	*P* value
Sex Male (vs. Female)	1.339	1.074–1.669	0.009	1.409	1.117–1.778	0.004
Age (years) ≥ 70 (vs. < 70)	2.351	1.919–2.880	< 0.001	1.816	1.458–2.262	< 0.001
BMI < 18.5 kg/m^2^ (vs. ≥ 18.5 kg/m^2^)	1.656	1.160–2.366	0.006	1.376	0.938–2.019	0.103
≥ 25.0 kg/m^2^ (vs. < 25.0 kg/m^2^)	0.874	0.690–1.109	0.268	0.998	0.775–1.285	0.985
ASA-PS ≥ 3 (vs. ≤ 2)	2.506	1.926–3.261	< 0.001	1.935	1.447–2.588	< 0.001
Preoperative albumin < 3.5 g/dL (vs ≥ 3.5 g/dL)	3.394	2.638–4.367	< 0.001	1.291	0.970–1.718	0.080
Preoperative CRP ≥ 0.5 mg/dL (vs. < 0.5 mg/dL)	2.263	1.750–2.927	< 0.001	1.046	0.790–1.384	0.753
Preoperative SMI r-low SMI* (vs. High SMI)	1.748	1.404–2.176	< 0.001	1.314	1.044–1.654	0.020
Surgical procedure TG (vs. Non-TG)	2.409	1.972–2.945	< 0.001	1.564	1.263–1.936	< 0.001
Surgical approach Open surgery (vs. Laparoscopy)	3.314	2.705–4.060	< 0.001	1.231	0.959–1.581	0.104
Lymph node dissection D2 (vs. D1 +)	2.326	1.896–2.852	< 0.001	0.776	0.598–1.006	0.055
pStage II (vs. I) III (vs. I)	2.864 9.093	2.160–3.799 7.214–11.46	< 0.001 < 0.001	3.591 10.74	2.584–4.991 7.523–15.34	< 0.001 < 0.001
Histological type Undifferentiated (vs. Differentiated)	1.146	0.940–1.398	0.178	1.208	0.979–1.492	0.079
Severe complication Present (vs. Absent)	2.363	1.742–3.206	< 0.001	1.571	1.147–2.152	0.005
Adjuvant chemotherapy Present (vs. Absent)	2.479	2.021–3.040	< 0.001	0.551	0.424–0.716	< 0.001

*Alb* albumin, *ASA-PS* American Society of Anesthesiologists-physical status, *CI* confidence interval, *CRP* C-reactive protein, *HR* hazard ratio, *SMI* skeletal muscle mass index, *TG* total gastrectomy ^*^r-low SMI: < 44.3 cm^2^/m^2^ for men, < 37.6 cm^2^/m^2^ for women

Table 3 shows the results of multivariate analysis for CSS and OCS. The r-low SMI was an independent predictor of poor CSS (HR 1.543, 95% CI 1.115–2.136, *P* = 0.009), but it was not an independent predictor of poor OCS (HR 1.179, 95% CI 0.844–1.648, *P* = 0.335).

**Table 3:** Results of the cause-of-death analysis

Variables	Cancer-specific survival						Other-cause survival					
	Univariate analysis			Multivariate analysis			Univariate analysis			Multivariate analysis		
	HR	95%CI	*P* value	HR	95%CI	*P* value	HR	95%CI	P value	HR	95%CI	P value
Sex Male (vs. Female)	1.127	0.832–1.527	0.442	1.353	0.981–1.867	0.066	1.626	1.171–2.257	0.004	1.500	1.065–2.113	0.020
Age(years) ≥ 70 (vs. < 70)	1.569	1.182–2.083	0.002	1.169	0.854–1.600	0.329	3.603	2.650–4.900	< 0.001	2.744	1.981–3.801	< 0.001
BMI < 18.5 kg/m^2^ (vs. ≥ 18.5 kg/m^2^)	1.414	0.821–2.436	0.212	1.511	0.903–2.531	0.116	1.414	0.821–2.436	0.212	1.357	0.759–2.429	0.303
≥ 25.0 kg/m^2^ (vs. < 25.0 kg/m^2^)	0.978	0.706–1.355	0.896	0.901	0.618–1.314	0.589	0.978	0.706–1.355	0.896	1.134	0.803–1.602	0.474
ASA-PS ≥ 3 (vs. ≤ 2)	2.005	1.334–3.012	< 0.001	1.858	1.179–2.927	0.008	3.134	2.213–4.440	< 0.001	2.029	1.385–2.972	< 0.001
Preoperative Alb < 3.5 (vs. ≥ 3.5 g/dL)	3.237	2.252–4.654	< 0.001	1.124	0.750–1.684	0.572	3.458	2.415–4.953	< 0.001	1.620	1.068–2.458	0.023
Preoperative CRP ≥ 0.5 (vs. < 0.5 mg/dL)	2.384	1.665–3.412	< 0.001	1.023	0.693–1.509	0.909	2.071	1.417–3.027	< 0.001	1.069	0.702–1.626	0.756
Preoperative SMI r-low SMI* (vs. High SMI)	1.485	1.110–1.986	0.008	1.543	1.115–2.136	0.009	1.027	0.756–1.397	0.864	1.179	0.844–1.648	0.335
Surgical procedure TG (vs. Non-TG)	2.979	2.244–3.955	< 0.001	1.571	1.163–2.123	0.003	2.005	1.499–2.681	< 0.001	1.608	1.183–2.185	0.002
Surgical approach Open (vs. Laparoscopy)	4.927	3.620–6.706	< 0.001	1.088	0.768–1.542	0.635	2.291	1.729–3.036	< 0.001	1.465	1.036–2.073	0.031
Lymph node dissection D2 (vs. D1 +)	4.143	2.995–5.732	< 0.001	0.627	0.433–0.908	0.014	1.317	0.994–1.747	0.055	0.871	0.611–1.241	0.445
pStage II (vs. I)	16.20	8.377–31.31	< 0.001	22.08	10.95–44.51	< 0.001	1.439	0.993–2.086	0.055	1.753	1.132–2.713	0.012
III (vs. I)	64.74	35.01–119.7	< 0.001	86.15	41.67–178.1	< 0.001	2.689	1.922–3.761	< 0.001	3.226	1.953–5.328	< 0.001
Histological type Undifferentiated (vs. Differentiated)	1.716	1.283–2.296	< 0.001	1.385	1.019–1.881	0.038	0.758	0.569–1.010	0.059	1.071	0.791–1.450	0.657
Severe complication Present (vs. Absent)	2.387	1.544–3.690	< 0.001	1.532	0.974–2.411	0.065	2.540	1.668–3.869	< 0.001	1.761	1.144–2.710	0.010
Adjuvant chemotherapy Present (vs. Absent)	5.608	4.163–7.555	< 0.001	0.644	0.455–0.910	0.013	0.933	0.659–1.321	0.696	0.375	0.241–0.584	< 0.001

*Alb* albumin, *ASA-PS* American society of anesthesiologists-physical status, *CI* confidence interval, *CRP* C-reactive protein, *HR* hazard ratio, *SMI* skeletal muscle mass index, *TG* total gastrectomy ^*^r-low SMI: < 44.3 cm^2^/m^2^ for men, < 37.6 cm^2^/m^2^ for women

### Predictive performance of the model

The discriminative ability of the SMI-based prognostic model for OS was assessed using the C-index. The univariate model with SMI alone yielded a C-index of 0.57 (95% CI 0.54–0.59), while the multivariate model adjusted for age, sex, tumor stage, and comorbidities achieved a C-index of 0.82 (95% CI 0.80–0.84).

## Discussion

This study focused on patient with gastric cancer who had normal SMI and identified the SMI cutoff value associated with poor OS after gastrectomy. The r-low SMI defined by this cutoff value was not only an independent poor prognostic factor for OS, but also an independent poor prognostic factor for CSS. The results of this study identified patients with even poorer long-term survival among those who did not meet the low-SMI definition we previously established [11], clarifying the cutoff values for pre-frail or pre-sarcopenic patient groups.

For patients with gastric cancer who do not meet the low-SMI definition, our cutoff value may identify patients with even poorer long-term survival after radical gastrectomy. This cutoff value does not define low muscle mass in sarcopenia or malnutrition criteria, but is an important value indicating presarcopenia or pre-malnutrition, which are pre-disease states. A previous report showed that patients with gastric cancer who did not experience low muscle mass preoperatively but did develop low muscle mass postoperatively had poorer long-term survival [10]. Therefore, perioperative management that anticipates postoperative reductions in muscle mass after gastrectomy is necessary. Establishing a cutoff value to define the presarcopenia stage in this study may enable the identification of patients at risk of developing postoperative sarcopenia or those requiring postoperative monitoring.

Among patients with gastric cancer having SMI within the normal range, those with lower muscle mass had a higher incidence of death from gastric cancer. One plausible explanation for this finding is that low muscle mass may make it more difficult to continue adjuvant chemotherapy, although this mechanism should be interpreted with caution. A systematic review reported that lower muscle mass is associated with more severe chemotherapy-related toxicities, making treatment continuation difficult [15]. Consistent with this, the discontinuation rate of adjuvant chemotherapy was significantly higher in the men’s r-low SMI group; however, this difference was not statistically significant in women. Moreover, detailed information on chemotherapy dose intensity was not available in our dataset. Adjuvant chemotherapy is a key factor in suppressing long-term recurrence in patients with advanced gastric cancer [16, 17], and it was an independent factor associated with improved CSS in the multivariate analysis of this study. The inability to administer or complete adjuvant chemotherapy may therefore constitute a risk factor for recurrence. At present, robust evidence on sex-specific prognostic effects of muscle mass in gastric cancer is lacking, and the sex-related pattern observed in this study should be regarded as hypothesis-generating and requires confirmation in future investigations.

Among patients with gastric cancer not classified as having low SMI, both men and women in the lower muscle mass group had poorer OS than those in the higher muscle mass group. This indicates that using appropriate cutoff values for both men and women can help identify patients with poorer long-term survival. Previous reports have shown that low skeletal muscle mass is associated with long-term survival in men; however, its impact is less pronounced in women [18, 19]. This may be significantly influenced by the cutoff value used to define low skeletal muscle mass. In this study, we not only determined the cutoff value using the training set but also validated it using the validation set. Therefore, the defined cutoff value for relatively low skeletal muscle mass may enable broader identification of patients with poorer OS.

This study provides cutoff values for patients classified as prefrail or presarcopenic in routine clinical practice. As it is a cutoff value based on CT scans used for cancer staging, it can be readily applied worldwide, particularly in Asia. Previous reports have shown that a three-week preoperative exercise and nutritional intervention program can significantly increase muscle mass [20]. We previously reported the definition of low muscle mass requiring preoperative intervention [11], and identified a preliminary group for this study. Postoperative monitoring of patients at risk of low muscle mass enables appropriate intervention. Further prospective studies are required to confirm the universality of our results. Moreover, further prospective studies are needed to determine whether setting a 3–4-week intervention period for increasing muscle mass prolongs OS in patients with low SMI.

The present study had several limitations. First, it was a retrospective study. A prospective multicenter study should be conducted to establish the universality of these results. Second, confounding factors such as comorbidities and nutritional status, may not have been adequately considered. Third, because our cohort included only Asian populations, it remains unclear whether the same cutoff values would be applicable in other countries or regions. Further research is needed to demonstrate universality across other countries and racial groups. Fourth, the SMI cut-off values used in this study may be subject to instability and limited generalizability. We derived these cut-offs using restricted cubic splines and then restricted the search range to the 20–50th percentiles, selecting the point at which the HR was maximized within this interval. Although the lower bound was chosen considering event rates and the clinical assumption that lower muscle mass is associated with worse survival, this procedure remains partially data-driven and the estimated cut-offs may be sensitive to the chosen range and sampling variability. In addition, this study was specifically designed to evaluate SMI-based stratification in gastric cancer patients without low SMI. To remain consistent with our previous work, we excluded patients with low SMI according to previously reported cut-offs. While appropriate for the study objective, this design may limit comparability with the prior cut-offs and restrict the generalizability of the new thresholds across the full spectrum of muscle mass. We attempted to address external validity by allocating patients by center and demonstrating that the proposed cut-offs could be applied across multiple institutions; however, prospective studies in independent populations are still needed to confirm their robustness and generalizability. Despite these limitations, the main strength of this study is that the cutoff values were based on CT scans, which are frequently used in routine clinical practice. We are confident that the results of this study can be readily implemented in medical institutions that adopt SMI. Another strength of our study is that patients were split into a training set and an independent validation set at the facility level, and the spline-derived SMI cut-off values were developed in the training set and evaluated in the validation set. Although this design does not constitute a fully external validation in a separate cohort, the use of a cluster-level split allows partial assessment of generalizability across different centers and may enhance the robustness of the proposed cut-off. Further research is needed to clarify the effects of postoperative monitoring and appropriate postoperative interventions on improving postoperative outcomes in patients with gastric cancer and r-low SMI.

## Conclusion

Among patients with normal SMI, those with lower SMI had poorer long-term survival after radical gastrectomy. In the lower muscle mass group, OS and CSS may be poorer. Further research is required to elucidate the effects of postoperative monitoring and appropriate postoperative interventions on oncological outcomes in patients with gastric cancer and potentially low skeletal muscle mass.

## Data Availability

The datasets generated and/or analyzed in the current study are available from the corresponding author upon reasonable request.
