# Correction: Identification of patients at risk for poor survival among those with normal skeletal muscle mass: a multicenter retrospective validation study in gastric cancer

**DOI:** 10.1007/s10120-026-01793-0

**Published:** 2026-08-21

**Authors:** Ryota Matsui, Kazuyoshi Yamamoto, Yoshiro Yukawa, Masayoshi Terayama, Yukinori Kurokawa, Daisuke Ichikawa, Yoshitomo Yanagimoto, Takashi Oshima, Souya Nunobe, Takashi Kamei, Hiroya Takeuchi, Yoshihiro Nabeya, Masaki Kaibori, Naoki Hiki, Hidetoshi Eguchi, Yuichiro Doki

**Affiliations:** 1 Department of Gastroenterological Surgery, The Cancer Institute Hospital of Japanese Foundation for Cancer Research, 3-8-31, Ariake, Koto-ku, Tokyo, 135-8550 Japan; 2 Department of Gastroenterological Surgery, Graduate School of Medicine, The University of Osaka, Suita, Japan; 3 Department of Gastroenterological Surgery, Osaka International Cancer Institute, Osaka, Japan; 4 First Department of Surgery, Faculty of Medicine, Graduate School of Medicine, University of Yamanashi, Kofu, Yamanashi Japan; 5 Department of Surgery, Toyonaka Municipal Hospital, Toyonaka, Japan; 6 Department of Gastrointestinal Surgery, Kanagawa Cancer Center, Yokohama, Japan; 7 Department of Surgery, Tohoku University Graduate School of Medicine, Sendai, Japan; 8 Department of Surgery, Hamamatsu University School of Medicine, Hamamatsu, Japan; 9 Division of Esophago‑Gastrointestinal Surgery, Chiba Cancer Center, Chiba, Japan; 10 Department of Surgery, Kansai Medical University, Hirakata, Japan; 11 Department of Upper Gastrointestinal Surgery, School of Medicine, Kitasato University, Sagamihara, Japan

**Correction to: Gastric Cancer** **https://doi.org/10.1007/s10120-026-01778-z**

In this article, the affiliation details for Authors "Hidetoshi Eguchi and Yuichiro Doki" were incorrectly given as 'Department of Gastroenterological Surgery, Cancer Institute Hospital, Tokyo, Japan' but should have been 'Department of Gastroenterological Surgery, Graduate School of Medicine, The University of Osaka, Suita, Japan'.

